# Ancient mtDNA Genetic Variants Modulate mtDNA Transcription and Replication

**DOI:** 10.1371/journal.pgen.1000474

**Published:** 2009-05-08

**Authors:** Sarit Suissa, Zhibo Wang, Jason Poole, Sharine Wittkopp, Jeanette Feder, Timothy E. Shutt, Douglas C. Wallace, Gerald S. Shadel, Dan Mishmar

**Affiliations:** 1 Department of Life Sciences and National Institute of Biotechnology (NIBN), Ben-Gurion University of the Negev, Beer-Sheva, Israel; 2 Department of Pathology, Yale University School of Medicine, New Haven, Connecticut, United States of America; 3 The Center for Molecular and Mitochondrial Medicine and Genetics (MAMMAG), University of California Irvine, Irvine, California, United States of America; 4 Nanogen, Inc., San Diego, California, United States of America; Sackler Institute for Comparative Genomics, American Museum of Natural History, United States of America

## Abstract

Although the functional consequences of mitochondrial DNA (mtDNA) genetic backgrounds (haplotypes, haplogroups) have been demonstrated by both disease association studies and cell culture experiments, it is not clear which of the mutations within the haplogroup carry functional implications and which are “evolutionary silent hitchhikers”. We set forth to study the functionality of haplogroup-defining mutations within the mtDNA transcription/replication regulatory region by *in vitro* transcription, hypothesizing that haplogroup-defining mutations occurring within regulatory motifs of mtDNA could affect these processes. We thus screened >2500 complete human mtDNAs representing all major populations worldwide for natural variation in experimentally established protein binding sites and regulatory regions comprising a total of 241 bp in each mtDNA. Our screen revealed 77/241 sites showing point mutations that could be divided into non-fixed (57/77, 74%) and haplogroup/sub-haplogroup-defining changes (i.e., population fixed changes, 20/77, 26%). The variant defining Caucasian haplogroup J (C295T) increased the binding of TFAM (Electro Mobility Shift Assay) and the capacity of *in vitro* L-strand transcription, especially of a shorter transcript that maps immediately upstream of conserved sequence block 1 (CSB1), a region associated with RNA priming of mtDNA replication. Consistent with this finding, cybrids (i.e., cells sharing the same nuclear genetic background but differing in their mtDNA backgrounds) harboring haplogroup J mtDNA had a >2 fold increase in mtDNA copy number, as compared to cybrids containing haplogroup H, with no apparent differences in steady state levels of mtDNA-encoded transcripts. Hence, a haplogroup J regulatory region mutation affects mtDNA replication or stability, which may partially account for the phenotypic impact of this haplogroup. Our analysis thus demonstrates, for the first time, the functional impact of particular mtDNA haplogroup-defining control region mutations, paving the path towards assessing the functionality of both fixed and un-fixed genetic variants in the mitochondrial genome.

## Author Summary

Mitochondria, the ‘power plant’ of the cell, have their own distinct genome (mtDNA), whose sequence varies among individuals around the globe. This variation, which was formed by the accumulation of mutations (variants) during the course of evolution, appears to alter the susceptibility to common complex diseases (such as Parkinson's disease and diabetes). However, since the accumulation of mtDNA mutations over time results in the formation of new combinations (genetic backgrounds), it is not clear which of the mutations are functional and which are “evolutionary silent hitchhikers”. Thus we aimed at assessing the functionality of mtDNA genetic variants, focusing on variants within the mtDNA regulatory region, hypothesizing that they could affect mtDNA activity and maintenance. We found that a variant defining mtDNA genetic background ‘J’ significantly increased the transcriptional efficiency and elevated mtDNA copy numbers in cells, as compared to other genetic backgrounds. Hence, mtDNA regulatory region variants can affect mtDNA maintenance, which may partially account for the involvement of this genetic background in disease susceptibility. Our analysis demonstrates, for the first time, the functional impact of a particular mtDNA variant that was fixed during evolution. Moreover, our findings underline the functionality of mtDNA variants in the evolutionary variable regulatory region.

## Introduction

Mitochondria are the major sources for cellular energy, through the process of oxidative phosphorylation (OXPHOS), and thus play a central role in cell life and death. Since mitochondrial DNA (mtDNA) encodes 13 essential proteins of the energy production apparatus and 24 key factors of their translation machinery (i.e. 22 tRNAs and 2 rRNAs) it is not surprising that numerous association studies have demonstrated the involvement of mtDNA genetic backgrounds (haplotypes, haplogroups) in complex human disorders as well as in selective events that transpired during human evolution [1]. Such implied functional potential of natural mtDNA variants has gained recent experimental support in human and murine cytoplasmic hybrids (cybrids) [2],[3]. Further support was provided by back-cross experiments in Drosophila and rat as well as by inter-populations crosses in *Tigriopus Americana* revealing that the interaction of mtDNA with the nuclear genetic background is under selective constraint [4],[5],[6]. This implies that mtDNA variants underlying population divergence have phenotypic consequences. Nevertheless, all the above mentioned studies analyzed complete haplotypes, thus masking the effects of single nucleotide changes within haplogroups.

How thus can one isolate the functional effect of specific mutations from their linked genetic background? Previously, we considered evolutionary sequence conservation to assess the functional importance of all common genetic variants in coding mtDNA, following the logic, that the more conserved the nucleotide position among species, the higher the functional importance [7],[8],[9]. This approach revealed that certain mutations that define human mtDNA lineages alter nucleotide positions to an equivalent degree of conservation as disease-causing mutations. Although predicting functional potential for coding region mutations, this observation cannot currently be tested experimentally, largely due to technical difficulties hampering site-directed mutagenesis in human mtDNA. This obstacle could, however, be partially overcome by cloning mtDNA-encoded genes, changing their genetic code so as to permit their translation on cytoplasmic ribosomes and then re-direct the nascent protein products to the mitochondria by exploiting introduced mitochondrial targeting sequences [10],[11],[12]. The success of this approach is limited to only certain mtDNA-encoded proteins. By contrast, allotopic expression of some mtDNA-encoded proteins was toxic to cells [11]. As such, this approach was not used to study the functionality of common variants. Moreover, haplogroup-defining mtDNA variants are mapped to protein and RNA-coding genes, as well as to non-coding regions, thus calling for alternative approaches to assess the functionality of non-coding mtDNA haplogroup-defining mutations.

In contrast to the mtDNA coding region, most mitochondrial regulatory elements (excluding the three conserved sequence blocks, CSBs) are subjected to many back-mutations [13],[14]. Moreover, sequences of mtDNA regulatory elements are much more flexible over the evolutionary time scale. Nevertheless, alterations of certain nucleotide positions with relatively poor evolutionary conservation were associated with phenotypes [15] suggesting that attributes other than evolutionary conservation should be considered to assess the functionality of mtDNA control region changes.

**Figure 1:**
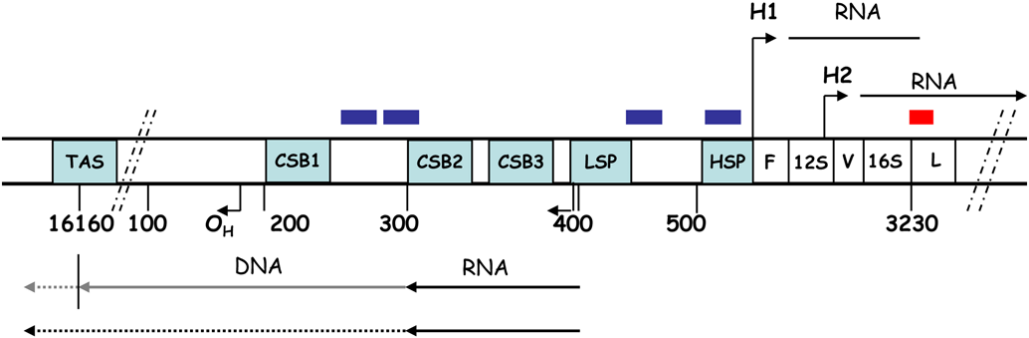
Schematic representation of the D-loop regulatory region. The names of regulatory motifs are indicated: the three conserved sequence blocks (CSB1, CSB2, and CSB3), light-strand promoter (LSP), heavy-strand promoter (HSP), the conserved termination-associated sequence (TAS), and origin of H-strand DNA replication (O_H_). Blue rectangles represent TFAM-binding sites and the red rectangle represents the mTERF binding site. The arrows pointing left and right show transcription orientation of H (H1 and H2) and L strands, respectively. The location of ribosomal RNA genes (12S and 16S) and the genes encoding tRNAs for phenylalanine, valine and leucine (F, V, and L, respectively) is indicated.

We hypothesized that certain sequence elements across the mitochondrial control region, not necessarily those with high inter-species conservation, are subjected to natural selection within populations. To test this hypothesis we chose to assess the functional potential of genetic variants in the mtDNA control region within sites harboring accepted functionality. mtDNA harbors sequence motifs that are recognized by members of the mtDNA transcription and replication machineries, all of which are encoded by the nuclear genome [16]. Such proteins bind particular sequences in the mtDNA promoters and origins of replication (Figure 1). Specifically, mitochondrial transcription factor A (TFAM) binds 4 mtDNA sites, two of which lay within the promoter region and two which lay down-stream to the L-strand promoter. The OXBOX-REBOX transcription factor binds a motif in CSB3 [14], while mitochondrial transcription termination factor (MTERF) recognizes a motif within tRNA-Leu (nucleotide positions 3232–3256) [17]. The termination-associated sequence (TAS) is bound by a specific protein that has yet to be isolated [18] and, finally, mtDNA origin of the light strand (Ori-L) is bound by specific factors (Joseph Shlomai, personal communication).

Here, we demonstrate that some single base changes that were fixed within TFAM binding sites during human evolution alter protein binding efficiency. One of these changes not only altered TFAM binding efficiency but also increased the efficiency of *in vitro* transcription by ∼2.5 fold and was associated with more than 2 fold increase in mtDNA copy numbers in mtDNA-less cell lines (rho-0 cells) repopulated with the mtDNA from haplogroup J. The interpretations of these results within the framework of understanding the implications of the phenotypic effects of haplogroup J are discussed.

## Results

We have screened more than 2500 whole mtDNA sequences from various human populations worldwide (http://www.genpat.uu.se/mtDB/) for natural variants in experimentally established protein binding sites and regulatory regions in the mtDNA control region (Table 1). These include recognition sites for mitochondrial transcription factor A (TFAM) and transcription termination factor (mTERF), the conserved sequence blocks (CSBs), and mitochondrial recognition sites for proteins of the replication machinery (TAS and origin of replication of the light strand - O_L_), comprising a total of 241 bp in each mtDNA. This screen identified that 77/241 of the total screened sites harbored polymorphic point mutations which can be divided into non-fixed changes (57/77, 74%) and haplogroups/sub-haplogroup-defining changes (i.e. population-fixed changes), comprising 20/77 (26%) of the variable nucleotide positions.

**Table 1:** Natural genetic variation in selected mtDNA regulatory elements.

Element	Total number	0 changes	1 change	2 changes	3 changes	4 changes	≥5 changes	Fixation events*
**CSB I (213–235)**	23	14	225,226,235	-	227	215	214,217,228,234	217-U5b225-X227-V1, W, X228-J1c, R31235-A
**CSB II (299–315)**	17	12	308,309,315	-	-	-	310,311	-
**CSB III (345–362)**	18	13	345,351,357,361	348	-	-	-	-
**Ori L (5730–5760)**	31	27	5741,5746	5747	5744	-	-	5744- B
**TFAM (233–260)**	28	11	235,237,241,242,243,248,250,255	246,252, 260	239,249,257	247	234,236	235-A247-L0/L1242-J1b250-I260-G2
**TFAM (276–303)**	28	20	281,282,285,291,298	280,284,295,297	-	279	-	280-D4285-U1295-J297-L1
**TFAM (418–445)**	28	25	418,431	-	-	437	-	431-D4
**TFAM (523–551)**	29	17	527,530,533,534,537,539,545, 547,549	546,548	-	-	-	-
**TAS (16158–16172**	15	3	16158,16160,16171	16163	16164,16170	16166	1616216167161681616916172	16162-F1/H116163-T116164-D516169-U5b16172-F1/N9a/U6/J4/I2
**mTERF (3232–3256)**	25	23	3253,3254	-	-	-	-	-

The columns represent the number of times that mutation events (change) occurred in a given nucleotide position.

*: represents fixed changes - mutations that were represented in more than 5 different mtDNA sequences belonging to the same phylogenetic branch (lineage, haplogroup). Letters within the ‘Fixation events’ column correspond to distinct human mtDNA haplogroups.

To assess the evolutionary conservation of the tested control region elements we have aligned the mitochondrial DNA (mtDNA) D-loop sequences of primates and other available mammalian sequences in each such sites (see Materials and Methods). Only four protein-binding sites described in human mtDNA gave trustworthy alignment (i.e. CSB1, CSB3, MTERF and OriL) and were, therefore, termed ‘highly conserved sites’ (HSS). The rest of our studied sites were either aligned only among humans and the great apes (i.e. chimpanzee, gorilla and orangutan) or within some primates, and were therefore regarded as ‘low conserved sites’ (LSS). It is important to note that CSB2 was excluded from the analysis as it is mapped to human mtDNA nucleotide positions 299–313 which mainly comprise a cytosine-tract prone to frequent insertions and deletions in humans, the frequency of which is hard to compute. We next documented the minimum number of changes that had occurred at each nucleotide position during mammalian evolution. Accordingly, nucleotide positions at HSS sites (n = 100) were divided into highly conserved positions, i.e. completely conserved positions (n = 54) and changeable positions (n = 46). While screening for human variants, we noticed a highly significant under-representation of variants within the highly conserved nucleotide positions (4/54), as compared to the evolutionary changed positions (16/46) (Fisher exact test, p = 0.0009). Thus, the more conserved the nucleotide position the less variable it is.

The LSS sites also showed a pattern of variability, with most nucleotides being invariable and most of the un-conserved nucleotide positions undergoing small number of changes in humans (Table 1). We believe that this pattern is, in fact, a reflection of variability in the functional importance of LSS positions, i.e. some nucleotide positions in these sites are functionally more important than others.

### Haplogroup J-defining mtDNA polymorphism alters TFAM binding and *in vitro* transcription

**Figure 2:**
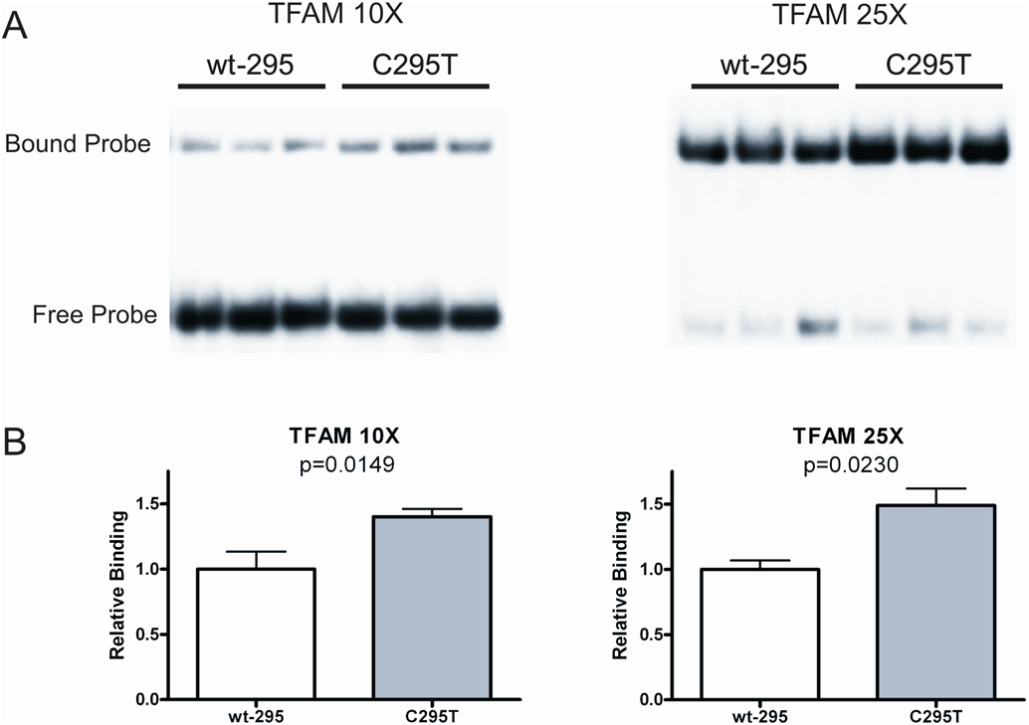
Increased TFAM binding to oligonucleotide probes harboring the C295T mtDNA variant. A. EMSA analysis of TFAM binding to double-stranded oligonucleotide probes containing the C295T mtDNA variant compared to its “wild-type” control (wt-295). Shown are autoradiograms of representative EMSA gels performed at a 10∶1 (TFAM 10×) and 25∶1 (TFAM 25×) TFAM∶probe molar ratio. The locations of the free and TFAM-bound (shifted) probe are indicated. B. Graphical representation and statistical analysis of the gels in A. The data of binding in the C295T variant is normalized to that of the wt-295, which was given a value of 1.0. The mean+/−one standard deviation (error bars) is shown as is the corresponding p-value.

**Figure 3:**
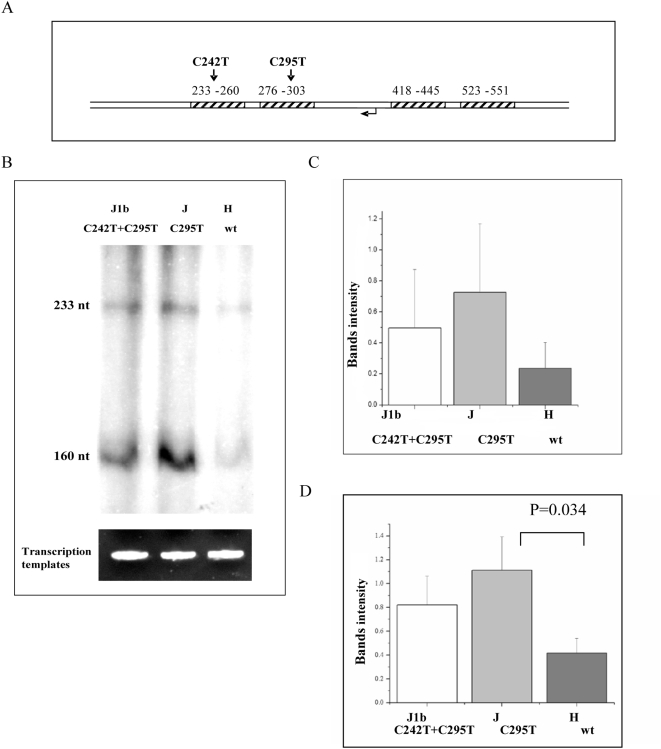
Run-off *in vitro* transcription assays preformed using mtDNA templates with or without TFAM binding site variants. A. A schematic map of the mtDNA templates used in the run-off *in vitro* transcription assay, with the TFAM-binding sites (striped rectangles), light-strand promoter (LSP, bent arrow), and the location of the 242 and 295 variants (arrows) indicated. B. A representative in vitro transcription reaction using templates containing the indicated mtDNA variants. Equal amounts of the linear mtDNA templates (ethidium bromide-stained at the bottom) were used in the *in vitro* transcription assay with a partially purified POLRMT (i.e. mtRNA polymerase) fraction from HeLa cells. The 223-nt full-length run-off transcript and a second major, shorter transcript of ∼160 nt (see Discussion) are indicated. Quantification of the 223-nt, full-length transcript and the ∼160-nt truncated product from multiple independent experiments is shown in C. and D., respectively. Both mutant templates showed a trend toward increased LSP transcription activity when either the full-length or shorter product was analyzed, but only in the case of shorter, ∼160 transcript from the haplogroup J template (C295T) was this difference statistically significant (p-value 0.034).

As the first step towards experimentally assessing the functional potential of haplogroup-defining mutations (fixed mutations), we employed Electrophoresis Mobility Shift Analysis (EMSA). Specifically, we tested for the effect of transition variants defining Caucasian haplogroup J (C295T) and a sub-haplogroup of J, J1b2 (C242T), on TFAM binding. These variants occur within two of the four TFAM-binding sites previously mapped by footprinting human mtDNA [19],[20]. EMSA analysis of double-stranded oligonucleotides spanning each of the two different TFAM binding sites, revealed increased TFAM binding to the C295T variant (Figure 2), but no significant difference with C242T variant (data not shown). In the case of the C295T variant, this increase in binding was observed at multiple different TFAM to DNA ratios (10/1 and 25/1 molar ratios are shown in Figure 2). These results raised the question of whether the C295T variant could affect mtDNA transcription. To test for this possibility, an mtDNA fragment spanning nucleotide positions 184–628 was PCR amplified using DNA samples corresponding to haplogroup J (harboring the C295T variant), haplogroup J1b2 (harboring both the C295T and C242T variants) and haplogroup H (harboring neither of these variants) as templates. The resulting PCR products were cloned and then utilized as templates in *in vitro* mitochondrial run-off transcription assays using partially purified mitochondrial lysates as a source of POLRMT and transcription factors [21]. The efficiency of light-strand promoter-driven transcription was ∼2.5 fold greater from the two haplogroup J templates (J, J1b2) than that of the haplogroup H fragment (H) (Figure 3), providing direct evidence for the functional potential of naturally occurring genetic variants in the human mtDNA regulatory region. Notably, the J1b2 construct showed increased transcription activity, although its additional mutation (C242T) did not significantly affect TFAM binding (data not shown), suggesting that the more functional variant is C295T that defines haplogroup J as a whole. Interestingly, the transcription assay produced two major products, namely a ∼230-nt, full-length transcript and a shorter ∼160-nt transcript that maps to just upstream of CSB1.

### mtDNA copy number is elevated in haplogroup J cybrids

**Figure 4:**
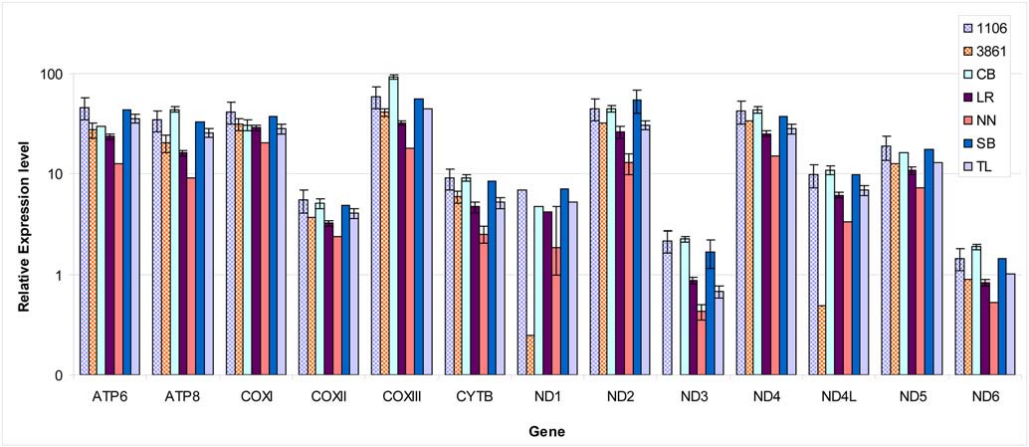
Relative steady-state levels of mtDNA transcripts in cybrids harboring different mtDNA haplogroups. Each color represents the transcription level estimated by real time quantitative PCR (see Materials and Methods) for mtDNA-encoded genes in the mentioned cybrids. Briefly, the measured transcripts levels were normalized to the geometric mean of the three housekeeping genes: GAPDH, β-Actin and β2 microglobulin. Cybrids haplogroup assignment was verified by PCR RFLP of selected polymorphic sites and sequencing and HVR1 and HVR2: cybrids 3861 and 1106 belong to haplogroup J1 and J1b2, respectively, while cybrids CB, LR, NN, SB and TL belong to haplogroup H. Notice that the transcript level of ND1 and ND4L genes were significantly lower in one of the haplogroup J1 cybrids (3861), and that the ND3 transcript level was lower in one of haplogroup H transcripts (NN).

**Figure 5:**
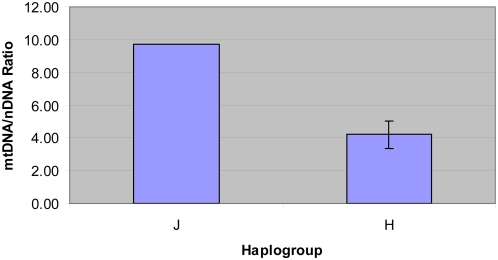
Increased mtDNA copy number in haplogroup J cybrids compared to haplogroup H cybrids. The copy number of mtDNA was measured using an mtDNA marker (ND2) and a nuclear DNA marker (18S rRNA gene). Data were obtained from three independent measurements for each of the tested cybrids, 2 of haplogroup J and 5 from haplogroup H. Standard deviation is shown for the mtDNA/nDNA ratios obtained from haplogroup J and haplogroup H cybrids.

We reasoned that our *in vitro* transcription results may not only reflect an effect of haplogroup J mutations on mtDNA transcription but also on replication, as these two processes are coupled in the mitochondria (reviewed in: [16]). Thus, in order to provide more clues to the physiological importance of our *in vitro* transcription findings, we assessed the effects of haplogroup J mutations on both mtDNA transcript levels (Figure 4) and mtDNA copy numbers (Figure 5) by real time PCR. To reduce variance in our measurements due to the expected effect of nuclear genetic factors, we performed our experiments in cytoplasmic hybrids (cybrids) carrying mtDNAs of haplogroup J (2 independent cybrids) or haplogroup H (5 independent cybrids). Two genes (i.e. ND1 and ND4L) exhibited reduced steady state levels in only one of the haplogroup J cybrids (designated 3861), whereas the other H- and L-strand transcripts showed no significance difference among the tested cybrids (Figure 4). Since steady state levels of mRNA reflect a combined effect of transcription and post-transcriptional regulation and since ND1 and ND4L are co-transcribed along with 10 H- strand genes (namely ND2-ND5, CO1-3, ATP6,8), the levels of which did not differ, we interpreted the lower levels of ND1 and ND4L in the 3861 cybrid as reflecting a post-transcriptional effect specific to this cybrid.

However, when the mtDNA copy number was assessed in the panel of cybrids, both haplogroup J cybrids had more than twice as many mtDNAs than did any of the haplogroup H cybrids (Figure 5). Since all cybrids were generated using the same Rho-0 cells (Wal-2A) and were grown under the same conditions, differences in the mtDNA copy numbers should reflect differences in the replication capacity of the different mtDNA haplotypes.

## Discussion

Our results reveal that some genetic variants that became fixed during human evolution affect protein binding to mtDNA. Moreover, a point variant in a TFAM binding-site (C295T) increased *in vitro* transcription ∼2.5 fold. These findings support the view that some evolutionarily fixed mtDNA variants lead to functional consequences. The C295T TFAM binding-site variant did not clearly align with other mammalian sequences, other than those of the great apes (humans, chimpanzees, gorillas and orangutans). Nevertheless, the C295T nucleotide position underwent only a single fixation event during human phylogeny in the branch leading to haplogroup J, suggesting that, although poorly conserved, the low human variability at this nucleotide position is in line with its functional potential. We thus suggest that the degree of variability of a nucleotide position within humans may serve as a clue for functionality. To experimentally test for the generality of this prediction the functionality of additional variable nucleotide positions should be tested.

Four TFAM-binding sites have been mapped in the human mitochondrial genome by DNA footprinting, two of which are important for transcription and are located between the heavy and light strand promoters. The importance of the other two binding sites, localized downstream to the light strand promoter is not known (Figure 1). The altered TFAM binding, as well as the altered *in vitro* transcription capacity of the C295T variant occurring in one of the downstream TFAM-binding sites, support the role of this site in mitochondrial transcription. To our knowledge, this is the first indication that TFAM binding downstream of the mtDNA promoter might influence transcription, possibly through a more complex configuration of the promoter or by modulating mtDNA wrapping by TFAM, as previously suggested [16].

The tested variants define known mtDNA haplogroups, namely Caucasian haplogroup J and a sub-group of haplogroup J (J1b2). Haplogroup J was previously associated with several complex phenotypes such as Parkinson's disease and longevity (reviewed in: [1] as well as with the tendency of type II diabetes patients to develop complications [22]. In addition, haplogroup J increases the penetrance of some mtDNA mutations causing the eye disorder, LHON (Leber Hereditary Optic Neuropathy). Since the C295T variant defines haplogroup J and was not fixed in any other human mtDNA lineage, it is possible that the observed functional effect can be partially attributed to its phenotypic effect. Since other mtDNA haplogroups have been associated with various health conditions, further analysis of haplogroup-defining variants will be revealing with regard to their impact on mitochondrial function and their potential health consequences.

In addition to the ∼230-nt, full-length transcription product expected in the run-off transcription assay, we observed a shorter ∼160-nt transcript (Figure 3) that extends to between CSB2 and CSB1, but closer to CSB1. The nature of this product is unclear, but it could be due to pausing or premature termination of transcription, processing of the transcript by RNAse [23] or due to a physiologically irrelevant nuclease activity in the extract used. Less than full-length products have been observed previously in human mitochondrial *in vitro* transcription assays using recombinant transcription components [24], however the main products observed in that study cluster near CSB2 not CSB1. Since the region surrounding CSB1 and CSB2 has been implicated in various aspects of transcriptional priming of mtDNA replication [24],[25],[26], and given the involvement of TFAM in mitochondrial transcription and replication [16], it is quite possible that, in addition to its effects on transcription documented herein, the C295T variant also could impact mtDNA replication efficiency. Using T7-based *in vitro* transcription experiment, a length polymorphism in mtDNA positions 303–315 was suggested to affect human mitochondrial transcription [27]. The elevated mtDNA copy number in haplogroups J versus H cybrids support the possibly increased replication capacity of haplogroups J. Since haplogroup J was associated with successful longevity in some European populations, and since mtDNA copy numbers are reduced over age, one can speculate that the elevated mtDNA copy numbers in haplogroup J may be one of the factors mediating the phenotypic effects observed for this haplogroup.

The increase in mtDNA copy number but not transcript levels in haplogroup J, as opposed to haplogroup H cybrids, raise the question of why differences in transcript levels were only observed cell-free *in vitro* transcription. Measurements of mtDNA transcript levels by real time PCR reflect the steady state level of mRNA products, which is affected by both transcriptional and post-transcriptional regulation. Therefore, it is still possible that direct measurement of transcription level *in organello* will reflect *in vitro* transcription differences between haplogroups J and H. Alternatively, it has recently been shown that increased copy numbers do not necessarily lead to increased transcription [28], suggesting that our *in vitro* transcription results reflect differences in replication rather than transcription, as discussed above.

Our experiments demonstrate the effects of specific point mutations in the mtDNA in an *in vitro* transcription assay. A major obstacle interfering with the analysis of specific mtDNA mutations is the current inability to perform site-directed mutagenesis within mtDNA. This could be partially overcome by mitochondrial import of mtDNA-encoded genes genetically engineering to be coded according to the cytoplasmic genetic code. Such re-coded mtDNA genes can be generated with or without the desired mutation. Indeed, this approach was successfully adopted for some mtDNA-encoded proteins (e.g. ATP6, ATP8, ND4 and ND2) but less successful with most proteins, implying limitations for this approach [10],[11],[12]. Such experiments are further complicated by the need to express the desired mutated mtDNA gene in cells not expressing the endogenous gene, a scenario currently available solely with natural disease-associated mutants. In contrast to coding region mutations, our experiments demonstrate that the effects of non-coding mtDNA mutations can be tested individually in cell-free systems.

In summary, our analysis sheds light on the functional effect of naturally occurring variants in the mtDNA control region and suggests that such variants contribute to the phenotypic impact of mitochondrial DNA haplogroups.

## Materials and Methods

### PCR and cloning of mtDNA control region fragments for *in vitro* transcription

DNA from healthy individuals previously assigned to haplogroups H, J1 and J1b2 [29], one sample of each, was used as a template for PCR amplification of an mtDNA fragment encompassing nucleotide positions 184–628 (using a forward primer: 5′GGCGAACATACTTACTAAAGTG 3′ and a reverse primer: 5′GCCCGTCTAAACATTTTCAG 3′). The PCR products were purified using the Wizard SV Gel and PCR Clean-up system (Promega) according to the manufacturer's protocol and then separately cloned into the pGEM T-vector (Invitrogen) using a ligation kit (Roche). The ligated products were transformed into *E. coli* strain DH5α by heat shock and ampicilin-resistant clones were isolated. Plasmid DNA harboring each of the cloned fragments was isolated and served as templates in an *in vitro* mitochondrial run-off transcription assay (see below).

### Electrophoretic mobility shift assays (EMSA)

Oligonucleotides corresponding to wild-type or mutant TFAM binding sites (Table 2) were annealed, followed by 5′ end labeling with Polynucleotide Kinase (NEB). Labeled DNA probes (3 nM) were incubated with varying amounts of TFAM protein (75 nM and 30 nM) for 30 minutes at room temperature in 10 mM Tris-HCL (pH 7.5), 10 mM KCl, 1 mM DTT, 1 mM EDTA, and 6% Glycerol. Free and TFAM-bound probes were then immediately separated on a 5% non-denaturing polyacrylamide gel at 150 V for 75 minutes in 0.5× TBE at room temperature. Gels were then dried, exposed to x-ray film and bands quantified using Quantity One 4.5.0 (BioRad) imaging software.

**Table 2:** List of double-stranded oligonucleotides containing the mtDNA TFAM-binding sites.

Name of the oligonucleotides	mtDNA nucleotide positions	Sequences
Wild type (CRS)	233–260	5′TAATAATAACAATTGAATGTCTGCACAG 3′
		3′ATTATTATTGTTAACTTACAGACGTGTC 5′
C242T–VAR (J1b)	233–260	5′TAATAATAA**T**AATTGAATGTCTGCACAG 3′
		3′ATTATTATT**A**TTAACTTACAGACGTGTC 5′
Wild type (CRS)	276–303	5′ACATCATAACAAAAAATTTCCACCAAAC 3′
		3′TGTAGTATTGTTTTTTAAAGGTGGTTTG 5′
C295T-VAR (J)	276–303	5′ACATCATAACAAAAAATTT**T**CACCAAAC 3′
		3′TGTAGTATTGTTTTTTAAA**A**GTGGTTTG 5′

Variants within two of the TFAM-binding sites (position 233–260 and 276–303) are underlined in bold. Type of mtDNA genetic background is shown in parentheses in the first column.

### *In vitro* transcription assay

Mitochondrial run-off transcription reactions were performed [21] using different mtDNA promoter-containing templates. PCR products corresponding to nucleotides 184–628 of human mtDNA and harboring mutations defining either haplogroup J, J1b2 or the Cambridge Reference Sequence (CRS) [30] were cloned into the plasmid pGEMT-EZ (Promega). Digestion of this plasmid with *EcoR1* resulted in the formation of a linear transcription template from which specific initiation from the LSP promoter resulted in transcripts 223 nucleotides in length. Individual reaction mixtures (25 µl) contained 36 µg of *EcoRI*-digested template, 10 mM Tris-Cl, pH 8.0, 10 mM MgCl_2_, 1 mM dithiothreitol, 100 µg/ml bovine serum albumin, 400 µM ATP, 150 µM CTP and GTP, 10 µM UTP, 0.2 µM α ^P32^UTP (3,000 Ci/mmol), 40 units of RNase OUTTM (Invitrogen) and 2.5 µl of a transcription-competent mitochondrial extract from HeLa cells that was prepared as described previously [21]. After 30 min at 30°C, reactions were stopped by adding 200 µl of stop buffer (10 mM Tris-Cl, pH 8.0, 0.2 M NaCl, and 1 mM EDTA). Samples were treated with 0.5% SDS and 100 µg/ml proteinase K for 45 min at 42°C and precipitated by adding 0.6 ml of ice-cold ethanol and 1 µg of yeast tRNA (Sigma). The resulting RNA pellets were dissolved in 20 µl of gel-loading buffer (98% formamide, 10 mM EDTA, pH 8.0, 0.025% xylene cyanol, 0.025% bromphenol blue), heated to 95°C for 5 min, and then separated on 6% polyacrylamide/7 M urea gels in 1× TBE buffer. Radiolabeled 10-bp ladder DNA (Invitrogen) was run in parallel as a marker to estimate RNA transcript sizes. Gels were dried and exposed to x-ray film at −80°C. The experiments were repeated 3 times.

### Databases

Human genetic variants were screened within experimentally established protein binding sites and regulatory regions in the mtDNA control region (Table 1). Specific nucleotide positions of the variants were retrieved from the “Human Mitochondrial Genome Database” (mtDB) (www.genpat.uu.se/mtDB), which includes more than 2500 whole mtDNA sequences, representing human populations worldwide. To assign control region variants to specific branches in the human mtDNA phylogeny, sequences containing genetic variants in the screened regulatory elements were compared to the revised Cambridge Reference Sequence [30]. Coding region variants in each of the retrieved sequences were used to screen the global human mtDNA phylogenetic tree that harbors worldwide human nucleotide variations, classified into haplogroups and sub-haplogroups [31]. Since mtDNA is transferred only through the maternal lineage and hence recombination has (virtually) no effect, this procedure enabled us to provide haplogroup assignments for mtDNAs harboring specific control region variants of interest that were linked to haplogroup-defining coding region mutations.

### Estimating the number of mutation events

We used a phylogenetic approach relying on haplogroup classification to determine the number of times that a mutation event at a given nucleotide position had occurred during human evolution. Such evaluation assumed that a group of sequences belonging to the same phylogenetic branch (i.e. lineage or haplogroup) represent one mutation event during human evolution. For fixed mutations, we set a cut-off value. Changes that were shared in 5 or more different mtDNA sequences belonging to the same phylogenetic branch were considered fixed. The reason for this cutoff value was, first, to avoid relatively recent fixation events that have yet to be statistically established as the number of whole mtDNA sequences increases. Secondly, branches harboring 2–4 sequences gave relatively low bootstrap values in the global mtDNA phylogenetic tree (data not shown). The conservation of mitochondrial regulatory elements in mammals was estimated using a multiple sequence alignment program [(MEME), http://meme.sdsc.edu/meme/intro.html]. The number of times that a mutation event occurred during the mammalian evolution was assessed using available phylogenetic trees (one for mammals and another tree for primates) [32],[33], assuming that mutations shared by members of the same tree branch had only occurred once in the ancestor of this branch.

### Lymphoblast cytoplasmic hybrids (cybrid) generation

The Wal2A rho zero nuclear donor cell line, which had been cured of its resident mtDNAs with ethidium bromide treatment [34], was fused to chemically enucleated lymphoblasts containing either haplogroup J or H mtDNA [35]. Donor lymphoblasts,1.5×10^6^ , were pre-treated with 0.5 µg/ml actinomycin D for 20 hours, the cells combined with 1.5×10^6^ Wal2A rho zero cells, and fused using a pH-balanced PEG-1450 mixture for 60 seconds. Cells were allowed a 5 day post-fusion recovery period in rho zero growth medium and then selected in a 1 µg/ml 6-thioguanine-supplemented DMEM medium containing 10% dialyzed serum solution for selection. Media were replaced every 4 days or when the media looked depleted until such time as the cells achieved normal growth rates, as determined by periodic cell counts. The cybrids were haplotyped post-fusion to determine successful transfer of the mtDNA. The presence of Wal2a nuclear background was confirmed by microsatellite testing and comparison to the original lymphoblast and Wal2A microsatellite identities. The haplogroup assignment of the cybrid clones was determined using haplogroup specific polymorphisms and by sequencing of the mtDNA control region [31].

### Determining expression levels of mtDNA-encoded genes in cybrids

mtDNA transcript levels were determined for each of the cybrids by PCR incorporation of SYBR Green dye into real time PCR products. The rate of dye incorporation was monitored using the Roche LightCycler 480 real-time PCR system. Target genes were normalized according to three reference gene products, namely GAPDH, β-Actin and β2-Microglobulin [36],[37]. The geometric mean of the reference genes *C_T_s* was used for normalization of transcript expression levels. Primers for the real time PCR reactions were designed using the Primer 3 software (Table 3). Four micro-liters of 5× SYBR Green PCR Master Mix (Roche) was mixed with 20 ng purified total RNA, 0.4 µM of gene-specific primers and ultra pure H_2_O in a 20 µl reaction volume within 384-well plates (Genesee Scientific and Roche). For amplification, reaction mixtures were incubated for 2 min at 50°C and 10 min at 95°C, followed by 45 cycles of three-steps consisting of 15 seconds at 95°C, 20 seconds at 60°C and 15 seconds at 72°C. Following the PCR step, samples were heated from 60°C to 95°C with a ramp time of 20 min to construct dissociation curves and verify that single PCR products were obtained. PCR products were also analyzed by gel electrophoresis to confirm primer specificity. Serial cDNA dilutions were used for primer validation experiments to demonstrate that both target and reference genes had equal amplification efficiencies, according to the standard curve method. The comparative *C_T_* method was used for relative quantification of gene expression as described by the real time PCR machine manual. Differences in the C*_T_* values (dC*_T_*) of the transcript of interest and reference genes were used to determine the relative expression of the gene in each sample. The dCT method was used to calculate fold expression. Experiments were carried out in triplicate for each data point. Roche analysis software versions 2.1 (Applied Biosystems) and Microsoft Excel were used for data analysis.

**Table 3:** Primers used for the real time PCR-based estimation of transcript levels and mtDNA copy numbers.

Primer name	Sequence
ND2 fwd	5′-ACTGCGCTAAGCTCGCACTGATTT-3′
ND2 rev	5′-GATTATGGATGCGGTTGCTTGCGT-3′
ND1 fwd	5′-ATGGCCAACCTCCTACTCCTCATT-3′
ND1 rev	5′-TTATGGCGTCAGCGAAGGGTTGTA-3′
Cox I fwd	5′-ACCCTAGACCAAACCTACGCCAAA-3′
Cox I rev	5′-TAGGCCGAGAAAGTGTTGTGGGAA-3′
CoxII fwd	5′-ACAGATGCAATTCCCGGACGTCTA-3′
CoxII rev	5′-GGCATGAAACTGTGGTTTGCTCCA-3′
ATP8 fwd	5′-ACCGTATGGCCCACCATAATTACC-3′
ATP8 rev	5′-TTTATGGGCTTTGGTGAGGGAGGT-3′
CoxIII fwd	5′-ACTTCCACTCCATAACGCTCCTCA-3′
CoxIII rev	5′-TGGCCTTGGTATGTGCTTTCTCGT-3′
ND3 fwd	5′-CCCTACCATGAGCCCTACAAACAA-3′
ND3 rev	5′-AGTCACTCATAGGCCAGACTTAGG-3′
ND4L fwd	5′-TATCGCTCACACCTCATATCCTCCCT-3′
ND4L rev	5′-AGGCGGCAAAGACTAGTATGGCAA-3′
ND4 fwd	5′-ACAAGCTCCATCTGCCTACGACAA-3′
ND4 rev	5′-TTATGAGAATGACTGCGCCGGTGA-3′
ND5 fwd	5′-ATCGGTTTCATCCTCGCCTTAGCA-3′
ND5 rev	5′-ACCTAATTGGGCTGATTTGCCTGC-3′
ND6 fwd	5′-AGGATTGGTGCTGTGGGTGAAAGA-3′
ND6 rev	5′-ATAGGATCCTCCCGAATCAACCCT-3′
CytB fwd	5′-TCCTCCCGTGAGGCCAAATATCAT-3′
CytB rev	5′-AAAGAATCGTGTGAGGGTGGGACT-3′
ATP6 fwd	5′-TAGCCCACTTCTTACCACAAGGCA-3′
ATP6 rev	5′-TGAGTAGGTGGCCTGCAGTAATGT-3′
12S fwd	5′-AAACTGCTCGCCAGAACACTACGA-3′
12S rev	5′-TGAGCAAGAGGTGGTGAGGTTGAT-3′
16S fwd	5′-TGTATGAATGGCTCCACGAGGGTT-3′
16S rev	5′-TAGGGTCTTCTCGTCTTGCTGTGT-3′
ND2 for	5′- CTAGCCCCCATCTCAATCATA -3′
ND2 rev	5′- GAATGCGGTAGTAGTTAGGAT -3′
18s S	5′- GAGAAACGGCTACCACATCC -3′
18s AS	5′- GCCTCGAAAGAGTCCTGTATTG -3′

Lines 1–30 (excluding column titles): primers used for Real Time PCR-based analysis of mtDNA transcripts levels in cybrids. Lines 31–34: primers used for Real Time PCR-based analysis of mtDNA copy number in cybrids.

### Assessment of mtDNA copy numbers in cybrids

mtDNA copy number calculations were performed by comparing the ratio of the mtDNA-encoded ND2 gene levels to the nuclear DNA-encoded 18S rRNA gene using real time quantitative PCR amplification (Roche LC480) in three independent experiments per tested DNA. To quantify each gene product, serial 10× dilutions of known cloned PCR fragments were used to create a standard curve. C*_T_* values of the ND2 and 18S rRNA genes were plotted against this standard curve to provide an absolute copy number level for each gene. The ND2 to 18S rRNA ratios were then calculated for each of the cybrid DNAs. Primers used for amplification of the ND2 and 18S rRNA genes are listed in Table 3.
